# Pancreatic surgery in elderly patients: results of 329 consecutive patients during 10 years

**DOI:** 10.3389/fmed.2023.1166402

**Published:** 2023-05-26

**Authors:** Emre Bozkurt, Emre Özoran, İbrahim Halil Özata, Çağrı Bilgiç, Mesut Kaya, Tutku Tüfekçi, Gürkan Tellioğlu, Orhan Bilge

**Affiliations:** ^1^Department of General Surgery, Koç University Hospital, Istanbul, Türkiye; ^2^Department of General Surgery, American Hospital, Istanbul, Türkiye

**Keywords:** pancreas, elderly, outcome, surgery, comorbidity

## Abstract

**Introduction:**

There is a progressive shift from a younger population to an older population throughout the world. With the population age shift, surgeons will be more encountered with older patient profiles. We aim to determine age-related risk factors of pancreatic cancer surgery and the effect of patient age on outcomes after pancreatic surgery.

**Materials and methods:**

A retrospective review was conducted with data obtained from consecutive 329 patients whose pancreatic surgery was performed by a single senior surgeon between January 2011 and December 2020. Patients were divided into three groups based on age: patients younger than 65 years old, between 65 and 74 years old, and older than 74 years old. Demographics and postoperative outcomes of the patients were evaluated and compared between these age groups.

**Results:**

The distribution of a total of 329 patients into the groups was 168 patients (51.06%) in Group 1 (age <65 years old), 93 patients (28.26%) in Group 2 (age ≥65 and <75 years old), and 68 patients (20.66%) in Group 3 (age ≥75 years old). The overall postoperative complications were statistically significantly higher in Group 3 than in Group 1 and Group 2 (*p* = 0.013). The comprehensive complication index of the patients in each group was 23.1 ± 6.8, 20.4 ± 8.1, and 20.5 + 6.9, respectively (*p* = 0.33). Fisher’s exact test indicated a significant difference in morbidity in patients with ASA 3–4 (*p* = 0.023). In-hospital or 90-day mortality was observed in two patients (0.62%), one from Group 2 and one from Group 3. The 3-year survival rates for each group were 65.4%, 58.8%, and 56.8%, respectively (*p* = 0.038).

**Conclusion:**

Our data demonstrate that comorbidity, ASA score, and the possibility of achieving a curative resection do have significantly more impact than age alone.

## 1. Introduction

There is a transition from a younger population pool to an aging generation throughout the world. For the first time in 2018, the world’s elderly population exceeded the population of children under the age of 5 years. People aged more than 65 years old represent the fastest-growing subset of the world population. The elderly (>65 years old) population constitutes 9.5% of the general population in Turkey (1). According to population projection estimates by the Ministry of Family, Labor and Social Services of the Republic of Turkey, the percentage of elderly people (age >65 years old) will be 16.3% of the population by 2040 and will reach up to 22.6% by 2060 in Turkey (1). This population change will lead to more confrontations with older patients. Due to this projection, the incidence of diseases that would require surgical treatment will be increased in elderly patients, and new research studies will focus on the application of current surgical practice on the elderly population safely.

The incidence and mortality of cancer cases increase with advanced age. More than half of patients with hepatobiliary malignancies are generally diagnosed after the age of 60 years (2). Although surgical resection is the only curative intended treatment option for pancreatic cancer, only 20% of pancreatic tumors are resectable at the time of diagnosis (3). Pancreatic cancer is one of the most malignant diseases, and its prognosis is dismal. The 5-year survival rate for this dreaded disease is 10%, and this rate decreases with increased patient age according to the SEER database (4). While the estimated mortality rate for pancreas cancer is 4.5 per 1,00,000 cases within all age groups, it increases to 53.3 per 1,00,000 over the age of 70 years (5). The increased mortality is not due to the surgical procedure alone but is mostly caused by the comorbidities of the patients (6).

Surgical resection using a radical approach is still the mainstay treatment option for pancreatic cancer. It is frequently mentioned in the literature that pancreatic surgery causes high mortality, high morbidity rates, and longer lengths of hospital stay in elderly patients (7–9). In contrast to this notion, some authors have reported that increased age is not a risk factor for postoperative mortality after pancreatic resection (10, 11). These opposing views make it difficult to make a conclusion about the safety of performing pancreatic surgery in the elderly population. Considering these data in the literature, there are some controversial points while planning surgical treatment of pancreatic diseases in the elderly patient group: (1) Is there a specified risk defined for pancreatic surgery in the elderly population? (2) Does increasing age increase mortality after pancreatic surgery? and (3) Does patient age affect postoperative outcomes in pancreatic surgery?

All these data suggest that dealing with pancreatic diseases in an elderly population can be challenging from the surgeon’s perspective, making it necessary to estimate the outcome of pancreatic resection in elderly patients for assisting in surgical decision-making. We aim to determine the age-related risk factors for pancreatic surgery and the effect of patient age on outcomes after pancreatic surgery.

## 2. Materials and methods

We conducted a retrospective review of the data of consecutive 329 patients who underwent pancreatic surgery performed by a single senior surgeon of the Vehbi Koç Foundation Hospitals (American Hospital and Koç University Hospital), Department of General Surgery, between January 2011 and December 2020. Patients with missing follow-up data were excluded from the study. The patients’ demographic data, Charlson Comorbidity Index, the American Society of Anesthesiologists (ASA) Physical Status Classification, hospital records, complications (bleeding, pancreatic fistula, bile leakage, and delayed gastric emptying), Clavien–Dindo grade ≥ 3a complications, comprehensive complication index, operative details (type of surgery, duration of surgery, and blood loss), pathology reports, length of hospital stay, readmission, and in-hospital or 90-day mortality status were recorded. This retrospective study protocol was approved by the Institutional Review Board and Ethics Committee of the Koç University Hospital (approval code: 2021.141.IRB1.045). All methods were carried out in accordance with the relevant guidelines and regulations of the Institutional Review Board.

Patients were divided into three groups: patients aged less than 65 years old (Group 1), between 65 and 74 years old (Group 2), and older than 74 years old (Group 3). The Charlson Comorbidity Index and data related to postoperative morbidity were available for all patients. Postoperative pancreatic fistula formation and delayed gastric emptying were defined according to the International Study Group of Pancreatic Surgery (12). Postoperative outcomes of the patients were evaluated and compared between the age groups.

The primary outcome was 90-day postoperative morbidity and mortality, and the additional outcomes include overall survival (OS), postoperative complications, pancreatic fistula formation, delayed gastric emptying, length of hospital stay, and histopathological results.

### 2.1. Statistical analyses

All analyses were performed using the Statistical Product and Service Solutions (SPSS) software package (version 21.0, SPSS-IBM, Armonk, NY, United States) at a 95% confidence level, and a *p-*value of < 0.05 was the statistically significant level. Data were obtained by a retrospective review of the maintained database. Quantitative variables were reported as the mean and standard deviation (SD); qualitative variables were described as numbers and percentages. One-way analysis of variance (ANOVA) was used to determine whether there were any statistically significant differences between the means of three or more independent (unrelated) groups. Differences between continuous and categorical variables were assessed using Student’s *t*-test for normally distributed variables and the Mann–Whitney *U*-test for non-normally distributed variables, and Fisher’s exact test or the chi-square test were used, respectively. We used Cox proportional hazard regression analysis to model the relationship between predictor variables and survival outcomes. This method allowed us to estimate hazard ratios and assess the significance of predictor variables such as age, ASA Physical Status Classification, comorbidity, blood loss, final histopathology, and TNM stage while accounting for censoring in the data. Overall survival was defined as the period between surgery and death and analyzed using the Kaplan–Meier method, and survival differences between ages were compared by using the Breslow test.

**Figure 1:**
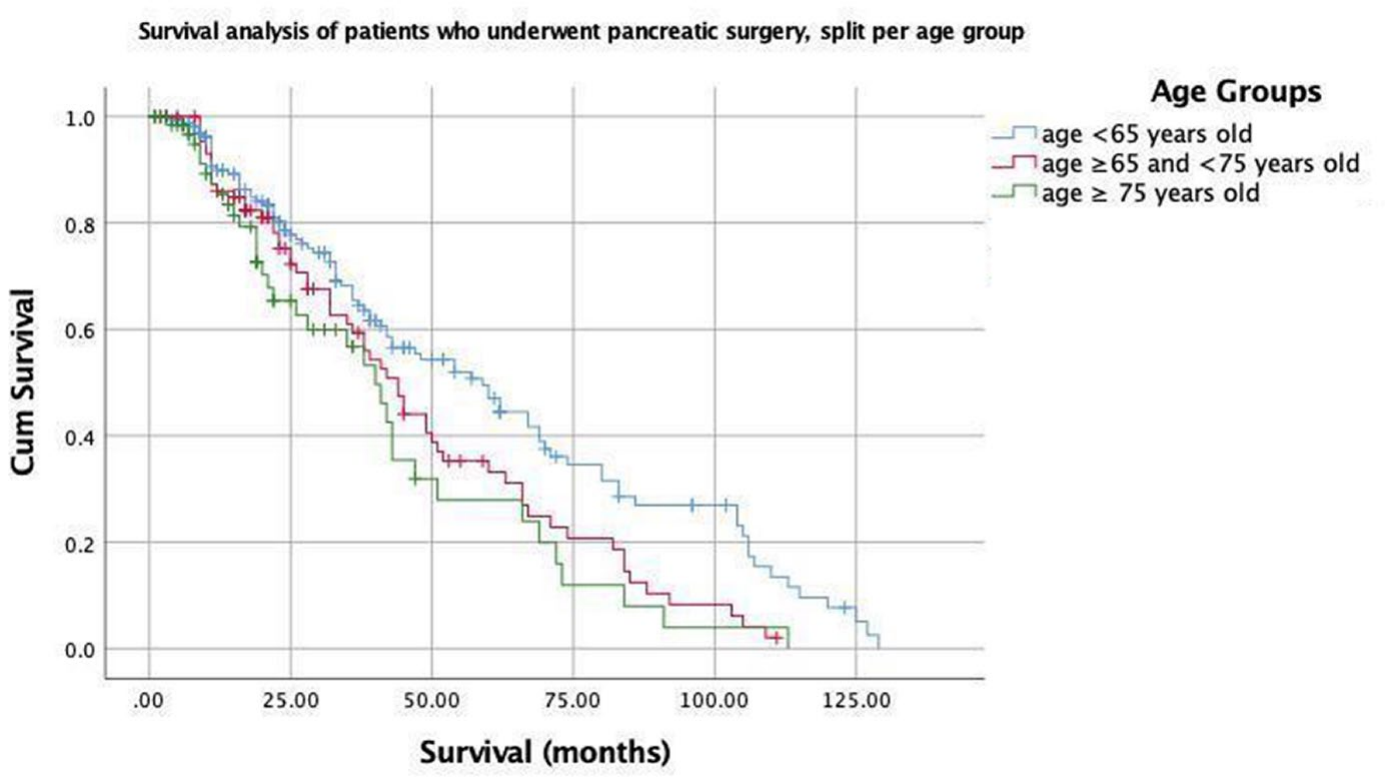
Kaplan–Meier curves for patients who underwent pancreatic surgery, split per age group (<65; ≥65 and  <75; and  ≥75  years old).

## 3. Results

The distribution of a total of 329 patients by age group was 168 patients (51.06%) in Group 1, 93 patients (28.26%) in Group 2, and 68 patients (20.66%) in Group 3. A total of two patients in Group 1, three patients in Group 2, and two patients in Group 3 were excluded from the study due to missing data. The remaining 322 patients with a female-to-male ratio of 1:1.2 were included in the final analysis. The mean ages of the patients in each group were 51.28 ± 9.55, 68.02 ± 2.76, and 78.64 ± 3.4 years. Several clinicopathological characteristics were analyzed to make comparisons between groups (Table 1). The distribution of the surgical procedures and histopathological diagnosis according to the groups is given in Table 1. Of these, 236 (73.3%) were pancreaticoduodenectomies, 33 (10.24%) were distal pancreatectomies, 15 (4.65%) were left pancreatectomies, 31 (9.62%) were total pancreatectomies, 4 (1.24%) were enucleations, 1 (0.31%) was a segmental pancreatectomy, and 2 (0.62%) were drainage procedures (Table 1). The mean operation time in each group was 295.6 ± 84.8; 306.77 ± 84, and 295.2 ± 68.1 min (*p* = 0.54). Operative data demonstrated that mean + SD blood loss was 250 ± 255, 343 ± 423, and 272 ± 239 mL in Groups 1, 2, and 3, respectively (*p* = 0.27).

**Table 1:** Characteristics of the patients.

	<65 years old	**≥**65 and <75 years old	**≥**75 years old	*p*-value
Sex, *n*				0.026
Male	79	54	43	
Female	87	36	23	
Age, mean **±** SD years old	51.28 ± 9.55	68.02 ± 2.76	78.64 ± 3.41	
ASA, *n*				<0.001
1–2	116	40	12	
3–4	50	50	54	
Charlson comorbidity index				<0.001
Median	3	5	7	
Range	0–8	1–8	5–9	
Procedure, *n*				0.12
Pancreaticoduodenectomy	121	59	56	
Distal pancreatectomy	26	3	4	
Left pancreatectomy	5	4	6	
Total pancreatectomy	14	11	6	
Enucleation	3	1	0	
Segmentary pancreatectomy	1	0	0	
Drainage procedures	1	1	0	
Operation time, mean **±** SD minutes	295.6 ± 84.8	306.77 ± 84	295.2 ± 68.1	0.54
Blood loss, mean **±** SD ml	250 ± 255	343 ± 423	272 ± 239	0.27

The final diagnoses based on histopathological examination of surgical specimens were adenocarcinoma in 233 patients, pancreatic cystic neoplasm in 31 patients (serous neoplasm in 10, mucinous neoplasm in 3, IPMN in 14, and solid pseudopapillary tumor in 4 of the patients), neuroendocrine tumor in 27 patients, mesenchymal tumors (gastrointestinal stromal tumor, myxoid neoplasia, and leiomyosarcoma) in 5 patients, metastasis in 4 patients, intra-ampullary papillary tubular neoplasm in 3 patients, sarcomatoid undifferentiated carcinoma in 2 patients, colloid carcinoma in 1 patient, lymphoma in 1 patient, and benign histopathology in 15 patients (Table 2). There was no difference in the distribution of benign patients between the groups (*p* = 0.14).

**Table 2:** Summary of postoperative course for all patients.

	<65 years old	**≥**65 and <75 years old	**≥**75 years old	*p*-value
In hospital or 90-day mortality, *n*				0.32
Yes	0	1	1	
No	166	89	65	
Length of hospital stay, mean ± SD	11.47 ± 5.22	11.38 ± 6.45	14.46 ± 8.14	0.018
Complications, *n* (%)	32 (19.3)	15 (16.7)	16 (24.2)	0.013
Delayed gastric emptying	7	6	7	0.33
Pancreatic fistula	19	6	5	
Bleeding	6	3	4	
Clavien-Dindo grade ≥ 3a, *n* (%)	7 (4.2)	6 (6.7)	7 (10.6)	
Comprehensive Complication Index, mean **±** SD	23.1 ± 6.8	20.4 ± 8.1	20.5 ± 6.9	
Histopathology, *n*				0.053
Adenocarcinoma	103	79	51	
Pancreatic cystic neoplasm				
Serous neoplasm	6	2	2	
Mucinous cystic neoplasm	3	0	0	
IPMN	9	3	2	
Solid pseudopapillary tumor	4	0	0	
Neuroendocrine tumor	20	5	2	
Mesenchymal tumor	5	0	0	
Metastasis	2	1	1	
Intraampullary papillary tubular neoplasm	1	1	1	
Sarcomatoid undifferentiated carcinoma	1	0	1	
Colloid carcinoma	0	0	1	
Lymphoma	0	1	0	
Benign histopathology	8	4	3	

Patients with ASA physical status classification of III and IV were more common in Group 3 (*n*1 = 50, *n*2 = 50, and *n*3 = 54 in Groups 1, 2, and 3, respectively, *p* < 0.001). The most common comorbidities were type 2 diabetes mellitus in Group 1, type 2 diabetes mellitus and ischemic heart disease in Group 2, and ischemic heart disease, type 2 diabetes mellitus, and dementia in Group 3. The Charlson Comorbidity Index was found to be statistically significantly high in Group 3 (*p* < 0.05). There was no statistically significant difference in terms of complications, morbidity, and mortality when comparing patients with the same ASA physical status between groups. When we divided the patients into two groups, ASA1-2 and ASA 3-4, Fisher’s exact test indicated that there was a significant increase in morbidity in patients with ASA 3-4 (*p* = 0.023).

The most commonly detected complications were clinically relevant pancreatic fistulas (*n* = 19, 11.44%), delayed gastric emptying (*n* = 7, 4.21%), and bleeding (*n* = 6, 3.61%) in Group 1; bleeding (*n* = 3, 3.33%), clinically relevant pancreatic fistulas (*n* = 6, 6.66%), delayed gastric emptying (*n* = 6, 6.66%), and pneumonia (*n* = 2, 2.22%) in Group 2; and delayed gastric emptying (*n* = 7, 10.6%), clinically relevant pancreatic fistulas (*n* = 5, 7.57%), bleeding (*n* = 4, 6.06%), and pneumonia (*n* = 2, 3.03%) in Group 3 (Table 2). The comprehensive complication index of the patients in each group was 23.1 ± 6.8, 20.4 ± 8.1, and 20.5 ± 6.9. While the postoperative complications were statistically significantly high in Group 3 (*p* = 0.013), there was no statistical significance between the comprehensive complication index of the patients in each group (*p* = 0.33). There was no statistically significant difference between the groups in terms of reoperation and readmission. The mean hospital stay of the patients in each group was 11.47 ± 5.22, 11.38 ± 6.45, and 14.46 ± 8.14 days, respectively (*p* = 0.018).

The median follow-up times of the patients were 13 months for all patients. Postoperative mortality within 90 days was observed in two patients (0.62%): one from Group 2 and one from Group 3. These two patients had a history of pancreaticoduodenectomy due to the diagnosis of pancreatic ductal adenocarcinoma for one patient in Group 2 and an ampullary tumor for one patient in Group 3. The Kaplan–Meier estimates indicated that the 3-year survival rates were 65.4, 58.8, and 56.8% for patients in Groups 1, 2, and 3, respectively, and the Breslow test indicated that there was a statistically significant difference between the survival rates (*p* = 0.038) (Figure 1). The mean survival times were 62, 48, and 43 months for patients in Groups 1, 2, and 3, respectively (Table 3). Cox multivariate analysis revealed that an ASA score of 3–4 was negatively correlated with survival and positively correlated with mortality (*p* < 0.001, 95% CI). Collectively, these results suggest that the elderly group survives for a shorter period of time because an ASA score of 3–4 is more commonly found in the elderly group.

**Table 3:** Survival characteristics of the study sample.

	Number of cases	Number of events	Number censored	Mean survival time in months (95% CI)
Group 1	166	89	77 (46.38%)	62 (54, 69)
Group 2	90	60	30 (33.3%)	48 (41, 55)
Group 3	66	35	31 (47%)	43 (34, 52)

## 4. Discussion

While the proportion of people over 75 years old constituted 2.45% of the population in 2010, it increased to 3.5% in 2020 (13). Due to the global increase in the elderly population, the proportion of patients who need surgical treatment after the 7th decade is increasing. As age is an independent risk factor for pancreatic cancer and the age distribution in Turkey is comparable with these global trends, the elderly population in need of pancreatic surgery is increasing in Turkey (14), but there are limited data on this subject in the literature. Therefore, the effect of age on the outcome after pancreatic surgery is the current interest of researchers for future projections. Although with the developments in surgical techniques and patient management, surgical procedures with high morbidity can be successfully applied to this particular patient group, pancreatic resections still carry up to 40% morbidity and up to 3.8% of mortality in high-volume centers (15). Talarico et al. (16) reported that the percentage of cancer patients aged ≥65, ≥70, and ≥ 75 years old was found to be 36, 20, and 9%, respectively, in clinical trials, and these rates make up almost half of the population that develops cancer. The fact that the patient group aged over 75 years represents 18% of this study group, which is higher than the reported studies in the literature makes our study even more valuable.

Complications reported after pancreatic resection are delayed gastric emptying and pancreatic fistula specific to resection, whereas complications related to surgery are pneumonia and bleeding (17, 18). While general complications after resection were statistically significant in Group 3, no statistically significant difference was found between Groups 1 and 3 in terms of pancreatic resection-related complications (Table 2). The occurrence of delayed gastric emptying after pancreatic surgery did not differ statistically between groups. The lower rate of pancreatic fistulas in Group 2 is due to the fact that the number of patients who underwent total pancreatectomies were more in this group. Although Ito et al. (19) reported higher rates of pancreatic fistula in the elderly group, our study concluded that there was no statistically significant difference in the occurrence of postoperative pancreatic fistulas in patients with advanced age. Post-pancreatic resection hemorrhage has been reported in up to 13.6% of patients in the literature (20). Postoperative bleeding was detected in 4.03% (*n* = 13) of the whole study group. Postoperative bleeding occurred more in Group 3 without reaching statistical significance as the proportion of ASA 3 and 4 patients with cardiac comorbidities was higher in these groups. Moreover, this study established that there was no statistically significant difference in terms of readmission and reoperation in different age groups.

Suzuki et al. (21) reported no differences between patient groups based on age in terms of morbidity and mortality. They reported that the prognosis of patients with higher ASA scores was worse in all age groups in subgroup analyses of the studies reporting the detrimental effect of advanced age on postoperative outcomes after pancreatic surgery (22, 23). The proportion of patients with high ASA scores in our study was higher in the elderly patient group, which resulted in the elderly patient group being more susceptible to complications (23). Complications that needed to be managed medically and length of hospital stays were significantly higher in elderly patients. These findings are controversial with the literature probably because of the higher proportion of ASA 3-4 patients in Group 3 and, although not statistically significant, the high rate of bleeding and pneumonia development in Group 3 increased the duration of hospital stays in elderly patients (11, 24). These findings suggest that the ASA score and Charlson Comorbidity Index are more important than patient age in predicting postoperative outcomes.

Some studies have reported that the prognosis after pancreatic surgery is poor for elderly patients older than 75 years old (25). Riall et al. (26) reported in their study, regarding the effect of age on survival in patients who underwent pancreatic resection, that they found no significant difference in terms of the effect of age on survival in five different age intervals (<70, 70–74, 75–79, 80–84, and > 85 years old). To reduce morbidity and mortality, Higuera et al. (27) suggested that accurate pre-anesthesia and cardiovascular risk assessment should be performed before the operation. In-hospital or 90-day mortality data did not achieve statistical significance in different age groups within this study. Our study supports the upper mentioned data with results of a negative correlation between the Charlson Comorbidity index and OS, and a higher postoperative bleeding rate in patients with high cardiovascular comorbidities. In addition to all these data, He et al. (28) reported that pancreatic surgery increases median and overall survival in elderly patients (>66 years old) when compared to patients who did not undergo surgical resection.

Surgical experience is related to improved postoperative outcomes. Feinglass et al. (29) portrayed that the impact of the surgeon’s experience is associated with improved postoperative outcomes after segmental colon resection. In our study, the surgeries were performed by a single senior surgeon, which could be the reason why the outcome of elderly patients and younger patients was comparable. This should be taken into consideration when evaluating the results of this study.

The main limitation of this study is its single-center retrospective design and lack of disease-free and cancer-specific long-term survival rates. Another limitation was that the oncological treatment reports were not included as the study included patients who had undergone pancreatic surgery for benign reasons. In addition, although the quality of life is an important criterion of studies in which age is the subject, it could not be included in the comparison parameters of this study due to its retrospective design. Quality of life studies may be conducted to eliminate the lack of data on this issue. Moreover, prospective multicenter studies with high sample sizes are still necessary to make conclusions about the effect of age on postoperative outcomes in elderly patients who had pancreatic surgery.

## 5. Conclusion

This study offers additional evidence that age alone should not exclude patients from the decision of having curative resection, but rigorous selection should be done. The fact that there is no difference in postoperative morbidity and mortality in patients with the same ASA score regardless of age makes pancreatic surgery safely applicable in particular elderly patients. The higher rate of complications in ASA 3 and 4 patients indicates that there should be more focus on the surgical decision-making of these patients. A high Charlson Comorbidity Index score is correlated with poor postoperative outcomes suggesting that comorbidity, ASA score, and the possibility of achieving curative resection are more important than age in patient selection for surgery.

## Data availability statement

The original contributions presented in the study are included in the article/supplementary material, further inquiries can be directed to the corresponding author.

## Ethics statement

This retrospective study protocol was approved by the Institutional Review Board and Ethics Committee of the Koç University Hospital (approval code: 2021.141.IRB1.045). Written informed consent from the patients/participants was not required to participate in this study in accordance with the national legislation and the institutional requirements.

## Author contributions

EB: substantial contributions to conception and design, analysis and interpretation of data, and drafting of the article. EÖ: substantial contributions to conception and design, data collection, and analyzing. İÖ: substantial contributions to conception and design and acquisition of data. ÇB: substantial contributions to conception and design, data collection, and drafting of the article. MK, TT, GT, and OB: substantial contributions to conception and design, acquisition of data, and drafting of the article. All authors contributed to the article and approved the submitted version.

## Conflict of interest

The authors declare that the research was conducted in the absence of any commercial or financial relationships that could be construed as a potential conflict of interest.

## Publisher’s note

All claims expressed in this article are solely those of the authors and do not necessarily represent those of their affiliated organizations, or those of the publisher, the editors and the reviewers. Any product that may be evaluated in this article, or claim that may be made by its manufacturer, is not guaranteed or endorsed by the publisher.
